# Trends in utilisation of plain X-rays by older Australians (2010–2019)

**DOI:** 10.1186/s12877-022-02786-1

**Published:** 2022-02-04

**Authors:** Virginie Gaget, Maria C. Inacio, David R. Tivey, Robert N. Jorissen, Wendy Babidge, Renuka Visvanathan, Guy J. Maddern

**Affiliations:** 1 Present Address: Surgical Specialities, University of Adelaide, The Queen Elizabeth Hospital, Woodville, SA 5011 Australia; 2 Registry of Senior Australians, South Australian Health and Medical Research Institute, Adelaide, SA 5001 Australia; 3 UniSA Allied Health and Human Movement, University of South Australia, Adelaide, Australia; 4 Royal Australasian College of Surgeons, Adelaide, SA 5001 Australia; 5 Adelaide Geriatrics Training and Research With Aged Care Centre (GTRAC), Faculty of Health and Medical Sciences, University of Adelaide, Woodville, SA 5011 Australia; 6 Aged & Extended Care Services, The Queen Elizabeth Hospital, Central Adelaide Local Health Network, Woodville, SA 5011 Australia

**Keywords:** X-ray, Radiology, Residential setting, Geriatrics

## Abstract

**Background:**

Older Australians are major health service users and early diagnosis is key in the management of their health. Radiological services are an important component of diagnosis and disease management planning in older Australians, but their national utilisation of diagnostic services has never been investigated in Australia.

**Purpose:**

This study aims to evaluate the utilisation of major plain X-rays by Australians ≥ 65 years old.

**Methods:**

A population-based epidemiological evaluation and yearly cross-sectional analyses of X-ray examinations per 1,000 Australians aged ≥ 65 years old between 2009 and 2019 were conducted using publicly available Medicare Benefits Schedule and Australian Bureau of Statistics data sources. Age and sex specific incidence rate (IR) of plain X-rays per 1,000 Australians, adjusted incidence rate ratios (IRR) and 95% confidence intervals (CI) were estimated using a negative binomial regression model.

**Results:**

During the study period, the Australian population over 65 years old increased by 39% while the crude plain X-ray utilisation by this population increased by 63%. Most X-rays were conducted on extremities or the chest. Men used chest radiography more than women, and particularly for lungs, where the incidence increased the most in those ≥ 85 years old. There was an increase in X-rays of extremities and the hip joint between 2009–10 and 2013–14 in people ≥ 85 years old.

**Conclusion:**

The utilisation of plain X-rays of the chest, the gastro-intestinal tract and extremities was high and has increased among older Australians between 2009–10 and 2018–19. Plain X-rays remain a commonly used diagnostic tool for conditions affecting the older population.

**Supplementary Information:**

The online version contains supplementary material available at 10.1186/s12877-022-02786-1.

## Background

With an increasingly ageing population, health care utilisation is rising, and the organisation of health services is evolving to better meet the needs of older people. In 2012, individuals aged 65 years and over represented 14% of the Australian population, a proportion predicted to increase to 18% by 2029 and 22% by 2061 [1, 2]. While most older Australians live at home without formal support, an increasing proportion of them receive government subsided aged care services, while living at home (22% of the Australian population ≥ 65 years old), or in residential aged care facilities (RACFs) (6% of the Australian population ≥ 65 years old) [3].

In Australia, people ≥ 65 years old are major users of health services, constituting a large proportion of total hospitalisations (41%), days spent in hospitals (48%), and other primary and secondary health care services.[4, 5]. As part of these services, timely radiology is key in allowing for early treatment and the best possible care. A study by Miller and collaborators reported that patients ≥ 65 years old represented 25.2% of all diagnostic imaging examinations during the 2004–05 Australian financial year [6]. The availability and access to radiological assessments for this demographic varies depending on patient frailty and are particularly challenging for patients with reduced mobility. Delivering diagnostic imaging at the place of residence of frail patients has the potential to reduce emergency department presentations, and in turn reduce the risk of many issues associated with hospital encounters, including the stress to the individual and potential hospital ‘ramping’ (i.e. waiting in the ambulance prior to emergency department admission) [7]. Plain X-rays were the diagnostic imaging test ordered the most by general practitioners across all age categories before ultrasounds with a rate of 4.5 orders per 100 encounters and 2.7 orders per 100 encounters respectively [6]. These X-rays were mostly conducted for fall-related or skeletal issues and for acute bronchitis or bronchiolitis, conditions commonly experienced by the elder population [8–12]. This report demonstrates that easily transportable diagnostic imaging techniques, such as plain X-rays and ultrasounds, are still utilised abundantly and could represent a first step in delivering diagnostic technology at the place of residence. While plain X-rays remain a staple for the diagnosis of conditions experienced by older citizens, little is known about the specific utilisation of this diagnostic imaging technique by older Australians.

To answer this knowledge gap and estimate the potential for future service utilisation, the present study aims to: 1) evaluate the yearly crude and adjusted incidence of plain X-ray utilisation for the purpose of diagnosing health issues commonly experienced by older people; 2) identify changes in the incidence rate of use for plain X-rays between 2009 and 2019 and 3) examine age or sex differences relating to this service utilisation.

## Methods

### Study design, data sources and study population

This population based epidemiological study and yearly cross-sectional analyses were conducted using publicly available data from the Medicare statistics website and the Australian Bureau of Statistics (ABS) from the 1st of July 2009 to the 30th of June 2019 [1, 13, 14]. Data are displayed by Australian financial year, which starts on the 1st of July of a given year and ends on the 30th of June of the following year. The study population was Australians ≥ 65 years old between the financial years 2009–10 and 2018–19. The Medicare data evaluated corresponds to X-ray services subsidised by the Australian Medicare Benefits Schedule. The MBS subsidises health services to all Australian citizens and residents since 1984 and it covers all diagnostic imaging evaluations (at least partially) ordered by a general practitioner or emergency department physician [15]. In 2014–2015, it was estimated that 91% of all diagnostic imaging evaluations were ordered by these providers. While it is possible that individuals access these services completely privately, it is unlikely they would do so as there is a financial disincentive for them to do so. The present evaluation did therefore not include X-rays conducted entirely privately.

### Variables

We identified and included all MBS items that correspond to the plain X-ray examination of the chest, the gastrointestinal tract (GI), extremities and the hip joint (See Supplementary Table 1 for all coding and data source references) [16–18]. MBS listings between 2010 and 2019 were examined to identify these codes. This data represents the total amount of X-ray services claimed through the MBS by each age-sex category between 2009–10 and 2018–19.

### Statistical analysis

X-ray use was grouped by body part. The data obtained was analysed using R version 4.0.3 [19]. Overall, age and sex specific incidence and 95% confidence intervals (CIs) of service utilisation per 1,000 people were calculated and adjusted using publicly available data from the ABS for each group. Yearly incidence results for the overall ≥ 65 years old population or each age-sex category were adjusted using the population statistics published by the ABS for each of these categories. Age and sex adjusted incidence rate ratios (IRRs) and 95%CIs estimating changes in incidence between 2009–10 and 2018–19 were calculated using a negative binomial regression model and from the MASS package, which was selected to accommodate overdispersion in the data. Given the marked differences in trends between certain years for some of the groups examined, the rate of utilisation for two time periods were estimated (2009–10 to 2013–14 and 2014–15 to 2018–19). A P-value of < 0.05 was considered statistically significant in all models.

## Results

**Fig. 1:**
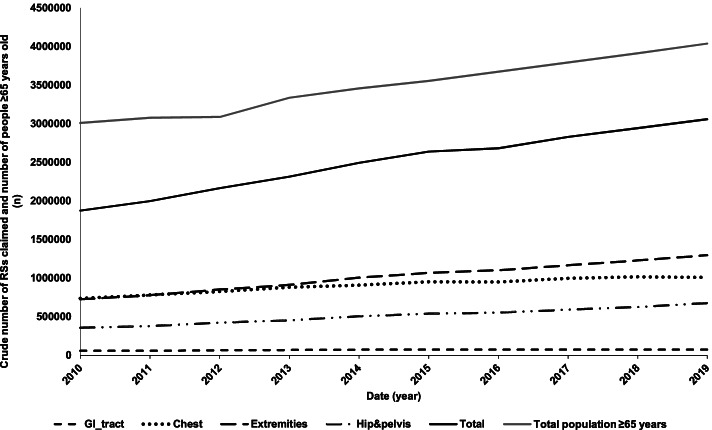
Trends in population growth and crude utilisation of plain X-rays of the GI tract, the chest, the extremities and hip and pelvis between 2009–10 and 2018–19

Between 2009–10 and 2018–19, the Australian population aged 65 years and over increased by 38% from 2,914,336 to 4,038,179 individuals (Fig. 1, Supplementary Table 2), with 2,146,438 women and 1,891,741 men over the age of 65 living in Australia in 2019 compared to 1,586,116 and 1,328,220 in 2010, respectively. The Australian population ≥ 65 years old represented 13% in 2010 and 16% of the total population in 2019.

### Total incidence of plain X-rays for the indication of fall-related injuries, pneumonia, heart failure and acute abdomen or bowel obstruction

In 2018–19, 3,058,677 plain X-rays of the chest, GI tract, extremities and the hip joint were conducted on patients ≥ 65 years old compared to 1,873,581 in 2009–10 (Fig. 1), representing a 63% increase in crude plain X-ray use. Most of these X-rays were performed on extremities (49%) and the chest (31%) (Fig. 1). More of these X-rays were conducted on women than on men, with 53% of crude incidence recorded for women in 2018–19, which is consistent with population composition, where females accounted for 53% of the population ≥ 65 years old.

### Changes in the incidence of utilisation rate of chest, GI tract and extremities X-rays over time and across age-sex groups

**Fig. 2:**
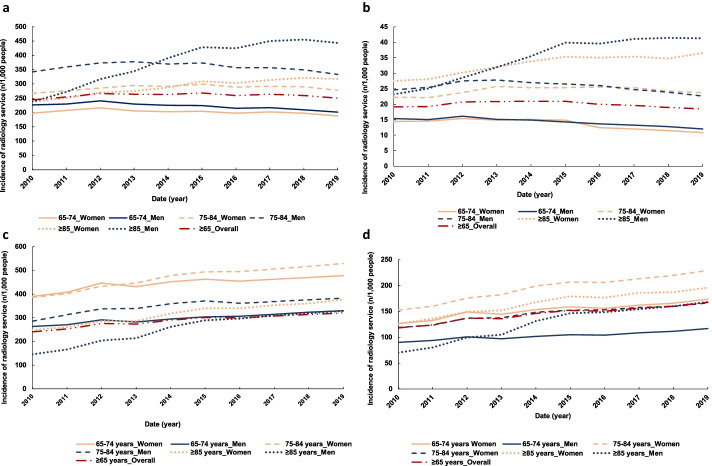
Trends in adjusted utilisation of plain X-rays by older Australians between 2009–10 and 2018–19 for the chest (**a**), the gastro-intestinal tract (**b**), extremities (**c**) and the hip and pelvis (**d**)

The overall adjusted incidence of plain chest X-rays was of 244/1,000 people ≥ 65 years old in 2009–10 versus 250/1,000 in 2018–19 (Fig. 2a). Plain lung X-rays accounted for 93% (range: 93%- 96%) of all chest radiographs conducted in 2018–19 across all age-sex groups. The adjusted incidence rate of plain chest X-rays was unchanged over time for the overall population ≥ 65 years old and most age-sex groups studied (Fig. 2a, Table1). Although most of these changes were not significant, the adjusted incidence of plain chest X-rays increased for all group evaluated between 2009–10 and 2013–14 before decreasing slightly between 2014–15 and 2018–19 (Table 1). The adjusted incidence of plain chest X-rays was higher in men, with the largest increase observed in men ≥ 85 years old, for which the incidence nearly doubled: 237/1,000 men in 2009–10 vs 443/1,000 men in 2018–19. (Fig. 2a). This represented a significant yearly utilisation increase (IRR: 1.13, 95%CI: 1.12–1.14) between 2009–10 and 2013–14. A smaller increase was also observed in women ≥ 85 years old between 2009–10 and 2013–14 (IRR: 1.05, 95%CI: 1.04–1.06).

**Table 1:** Adjusted incidence rate ratio of change in radiological service utilisation by Australian people aged 65 years and older overall and by age and sex group during two periods: 2009–10 to 2013–14 and 2014–15 to 2018–19

	Overall	Females			Males		
	≥ 65 years	65–74 years	75–84 years	≥ 85 years	65–74 years	75–84 years	≥ 85 years
***Radiology***							
Total chest							
2010–2014	1.04 (1.02–1.05)	1.00 (0.99–1.02)	1.02 (1.02–1.03)	1.05 (1.04–1.06)	1.00 (0.98–1.01)	1.02 (1.01–1.03)	1.13 (1.12–1.14)
2015–2019	0.99 (0.98–1.00)	0.98 (0.97–0.99)	0.99 (0.98–0.99)	1.01 (1.00–1.02)	0.98 (0.97–0.98)	0.98 (0.97–0.98)	1.01 (1.00–1.03)
Lung							
2010–2014	1.04 (1.02–1.05)	1.00 (0.99–1.02)	1.03 (1.02–1.03)	1.05 (1.04–1.06)	1.00 (0.98–1.01)	1.02 (1.01–1.03)	1.13 (1.12–1.14)
2015–2019	0.99 (0.98–1.00)	0.98 (0.97–0.99)	0.99 (0.98–0.99)	1.01 (1.00–1.02)	0.98 (0.97–0.98)	0.98 (0.97–0.98)	1.01 (1.00–1.03)
GI-tract							
2010–2014	1.04 (1.03–1.05)	1.01 (1.00–1.02)	1.04 (1.03–1.06)	1.06 (1.05–1.06)	0.99 (0.98–1.01)	1.03 (1.01–1.05)	1.12 (1.11–1.13)
2015–2019	0.97 (0.96–0.99)	0.93 (0.91–0.95)	0.98 (0.97–0.99)	1.01 (1.00–1.02)	0.96 (0.95–0.97)	0.96 (0.96–0.97)	1.01 (1.00–1.02)
Total extremities							
2010–2014	1.06 (1.05–1.08)	1.04 (1.02–1.05)	1.05 (1.05–1.06)	1.06 (1.05–1.07)	1.03 (1.01–1.04)	1.06 (1.04–1.07)	1.15 (1.13–1.17)
2015–2019	1.02 (1.02–1.02)	1.01 (1.00–1.02)	1.02 (1.01–1.02)	1.03 (1.02–1.03)	1.02 (1.02–1.02)	1.01 (1.00–1.02)	1.03 (1.03–1.04)
Upper extremities							
2010–2014	1.06 (1.05–1.08)	1.04 (1.02–1.05)	1.06 (1.05–1.06)	1.06 (1.05–1.08)	1.03 (1.02–1.05)	1.05 (1.04–1.07)	1.15 (1.12–1.17)
2015–2019	1.03 (1.02–1.03)	1.01 (1.01–1.02)	1.02 (1.02–1.03)	1.03 (1.03–1.04)	1.02 (1.01–1.02)	1.02 (1.02–1.03)	1.05 (1.04–1.06)
Lower extremities							
2010–2014	1.07 (1.05–1.08)	1.04 (1.02–1.05)	1.06 (1.05–1.07)	1.07 (1.06–1.08)	1.03 (1.01–1.04)	1.06 (1.05–1.08)	1.16 (1.14–1.18)
2015–2019	1.02 (1.01–1.02)	1.01 (1.00–1.01)	1.02 (1.01–1.02)	1.03 (1.02–1.04)	1.03 (1.02–1.03)	1.00 (0.99–1.02)	1.03 (1.02–1.03)
Total hip joint							
2010–2014	1.07 (1.06–1.09)	1.05 (1.03–1.07)	1.07 (1.06–1.08)	1.07 (1.06–1.08)	1.03 (1.01–1.04)	1.06 (1.04–1.07)	1.16 (1.14–1.19)
2015–2019	1.03 (1.02–1.03)	1.02 (1.01–1.03)	1.03 (1.02–1.03)	1.02 (1.01–1.03)	1.03 (1.02–1.04)	1.02 (1.02–1.03)	1.04 (1.03–1.04)
Pelvis							
2010–2014	1.10 (1.08–1.12)	1.08 (1.06–1.11)	1.11 (1.09–1.12)	1.11 (1.09–1.12)	1.04 (1.03–1.06)	1.09 (1.07–1.10)	1.19 (1.17–1.22)
2015–2019	1.04 (1.04–1.05)	1.04 (1.03–1.06)	1.05 (1.04–1.06)	1.04 (1.03–1.05)	1.05 (1.03–1.06)	1.04 (1.03–1.05)	1.05 (1.05–1.06)
***Hip fractures***							
2010–2014	1.01 (1.00–1.02)	1.02 (1.00–1.04)	1.01 (1.01–1.02)	0.99 (0.97–1.00)	1.01 (0.99–1.02)	1.01 (1.00–1.02)	1.03 (1.00–1.05)
2015–2018	1.01 (1.00–1.01)	1.01 (0.99–1.03)	1.01 (1.00–1.01)	0.98 (0.97–0.99)	1.02 (1.00–1.04)	1.01 (0.99–1.04)	1.00 (0.99–1.02)
***Hip surgeries***							
2010–2014	1.03 (1.02–1.04)	1.04 (1.01–1.06)	1.03 (1.02–1.04)	1.01 (1.00–1.03)	1.01 (0.99–1.02)	1.02 (1.01–1.03)	1.08 (1.05–1.11)
2015–2018	1.02 (1.01–1.03)	1.01 (0.99–1.04)	1.03 (1.03–1.04)	1.03 (1.01–1.05)	1.02 (1.00–1.04)	1.02 (0.99–1.05)	1.02 (1.00–1.04)

Abbreviations: *GI* gastro-intestinal

Note: All estimates were adjusted by age and gender and 95% confidence intervals are given in parentheses. Total chest = Lung + other thoracic examinations, Total extremities = Upper extremities + Lower extremities, Total hip joint = Hip + Pelvis, Hip fractures = all hip repairs associated with fractures as presented in the MBS and ACHI systems, and Hip surgeries = hip repairs not associated with fractures as presented in the MBS and ACHI systems

The crude and adjusted incidence in utilisation of GI tract X-rays did not change significantly over time for most age-sex groups examined, with an overall adjusted incidence of 19/1,000 people in 2009–10 compared to 18/1,000 people in 2018–19 (Fig. 2b, Table 1). In 2018–19 the incidence of plain GI tract X-rays was higher in people ≥ 85 years old with 36/1,000 people and 41/1,000 people plain abdominal X-rays conducted in women and men, respectively (Fig. 2b). This change corresponded to a significant yearly increase (IRR: 1.06, 95%CI: 1.05–1.06) between 2009–10 and 2013–14 in women ≥ 85 years old (Table 1). In comparison, men of the same age category saw a higher increase (IRR: 1.12, 95%CI:11–13%) over the same period. A yearly decrease (IRR: 0.93, 95%CI: 0.91–0.95) in utilisation of plain GI tract X-rays was observed in women aged 65–74 years old between 2014–15 and 2018–19. Men and women between 65 and 84 years old used these services similarly between 2009–10 and 2013–14, while the yearly increase was higher in men between 2014–15 and 2018–19.

A total of 240/1,000 people ≥ 65 years old received a plain X-ray of an extremity in 2009–10 versus 321/1,000 people in 2018–19 (Fig. 2c), while the overall utilisation did not change over time (Table 1). The incidence of these X-rays was higher in women, particularly in those 75 to 84 years old, while it was lower in those ≥ 85 years old (Fig. 2c). There was an increase in utilisation for those 75–84 years and over 85 years old with a significant yearly increase in women (IRR: 1.05, 95%CI: 1.05–1.06 and IRR: 1.06, 95%CI: 1.05–1.07) and in men (IRR: 1.06, 95%CI: 1.04–1.07 and IRR: 1.15, 95%CI: 1.13–1.17) in men respectively between 2009–10 and 2013–14 (Table 1). This increase was observed with services related to upper extremities both for women (IRR: 1.06, 95%CI: 1.05–1.08) and for men (95%CI: 4–7% and 12–17%) for men. Similarly, this trend was observed for lower extremities with a significant yearly increase for women (IRR: 1.06–1.07, 95%CI: 1.05–1.08) and for men (IRR: 1.06–1.16, 95%CI: 1.05–1.08% and 1.14–1.18).

Similarly, the incidence of X-rays of the hip joint increased from 118/1,000 people ≥ 65 years old in 2009–10 to 167/1,000 people in 2018–19 (Fig. 2d). The overall adjusted utilisation of pelvic X-rays increased the most, with a significant yearly increase (IRRs: 1.04 to 1.19, 95%CI: 1.03–1.06 to 1.17–1.22) observed in most age-sex categories between 2009–10 and 2013–14 (Table 1). The incidence of hip joint X-rays in those aged ≥ 65 years old increased, with a significant yearly increase (IRR: 1.05, 95%CI: 1.04–1.05) before slowing down in all categories from 2014–15 (Table 1, Fig. 2d). This increase in incidence between 2009–10 and 2013–14 was particularly high in men ≥ 85 years old (IRR: 1.16, 95%CI: 1.14–1.19) compared to women of the same age (IRR: 1.07, 95%CI: 1.06–1.08). The other age-sex categories had similar yearly changes (Table 1).

## Differences in utilisation between sex and age group for lung plain X-rays

**Fig. 3:**
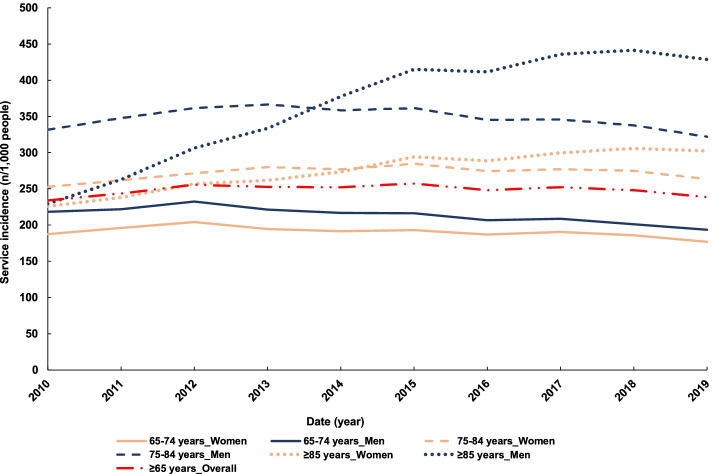
Trends in lung X-ray utilisation by older Australians between 2009–10 and 201,819

The adjusted use of plain lung X-rays increased in men ≥ 85 years old, with 229 examinations/1,000 men in 2010 and 428 examinations/1,000 men in 2019. In this population there was a significant yearly increase in utilisation between 2009–10 and 2013–14 (IRR = 1.13, 95%CI: 1.12–1.14) before reaching stabilisation in the second half of the decade (Fig. 3, Table 1). A similar pattern was observed in women ≥ 85 years old with a lower yearly increase (IRR: 1.05, 95%CI: 1.04–1.06) between 2009–10 and 2013–14. In each age category, men were utilising plain lung X-rays considerably more than women (Fig. 3). In 2018–19 a total of 42% of men ≥ 85 years old had a chest X-ray done versus 30% of women of the same age (if one examination is attributed to one individual). Similar trends were observed in the other age categories with 32% of 75 to 84 years old men having a plain chest X-ray done compared to 26% of women of the same age, and 19% of men versus 17% of women between 65 to 74 years old having a chest x-ray in 2018–19.

## Discussion

While the Australian population ≥ 65 years old has increased by 38% between 2009–10 and 2018–19, there has been a crude 63% increase in X-ray claims for this population. Patients ≥ 65 years old remain the largest users of diagnostic imaging, representing 38.5% of all diagnostic imaging examinations in 2021 [20]. This increase in X-ray use was driven by a greater number of X-rays performed on the chest and extremities, which is in line with the increase reported by other countries [21–23]. A study in 2005 reported that diagnostic radiology was the medical imaging technique most claimed in Australia with 4.5 examinations/100 encounters followed by ultrasound with 2.7 examinations/encounter and computed tomography (CT) with 1.0 examinations/encounter [6]. This can explain the crude increase of utilisation of this service as the population is ageing.

Specific X-rays have experienced varying utilisation trends over the period examined. The utilisation of X-rays of the GI tract increased nationally between 2009–10 and 2013–14, though followed by a downward trend. Recent studies reported on this type of service and found a 9% increase in the use of these services between 2000 and 2016 [23, 24]. It is likely that the downward trend observed after the initial increase is related to the limited diagnostic capacity of plain X-rays for abdominal issues compared to other techniques such as CT or ultrasound, therefore being superseded by those modalities [25]. This observation is confirmed by previous findings, where abdominal pain was listed in the top four causes for CT prescription (3.3% of CT) but did not appear in the top ten causes for X-ray referral (< 1.9% or X-rays) [6].

A significant increase in the incidence of plain chest X-rays was observed in people ≥ 85 years old, especially in men for who the incidence almost doubled over the decade evaluated. The increase was predominantly driven by lung X-rays. As the size of this group is smaller, the effect may be slightly amplified in the analysis compared to the other age groups. Acute bronchitis and bronchiolitis were listed as the 8^th^ most common condition for all plain X-rays ordered in 2004–05 overall in Australia [6]. Additionally, chest x-rays are subsidised through the mobile service for the diagnosis of pneumonia [26]. This indicates that chest infection is a condition commonly encountered by older Australians that require diagnostic imaging. Plain chest and lung X-rays remain, the gold standard diagnostic imaging tool to diagnose chest infections, in particular for pneumonia, which accounts for up to 40% of hospitalisations in the older population [11, 12, 16]. In 2012 it was reported that for the financial year 2011–12, 13 per 1,000 people ≥ 65 years old and 35 per 1,000 people in Australians ≥ 85 years old were hospitalised each year in due to pneumonia [10]. It is therefore not surprising that the incidence of chest X-rays increased over time in the older population. Additionally, while plain lung X-rays are used for the diagnosis of chest infections, they are also key for diagnosing lung cancer and other pulmonary obstructive diseases, both related to smoking and occupational exposure [27]. Historical statistics show a significant difference in smoking habits between men and women. In 1945, the time the ≥ 85 years old people in the present cohort turned 18 years old, 72% of men and 26% of women were regular smokers in Australia, which, in addition to occupational risk without appropriate occupational health and safety safeguards in place, could explain the differences observed between sexes in relation to lung X-ray utilisation [28]. While the difference in smoking reduced over time to reach 45% of men vs 30% of women in 1974, the impact of smoking-related negative health outcomes is expected to be seen for several decades [27, 29]. The downturn over the second part of the decade could be explained by a combination of (1) people who were younger and therefore less exposed to smoking into this age group and (2) the availability of improved treatment and management strategies for chronic obstructive pulmonary diseases. The noticeable increase in chest X-ray utilisation by people ≥ 85 years old demonstrates that this service is still largely used for diagnosing lung conditions in the elderly.

A significant increase in utilisation of X-rays of extremities was observed in people ≥ 75 years old. Contrary to chest X-rays, X-rays of extremities were used more frequently by women than men in those ≥ 65 years old, with a higher incidence in women aged between 75 and 84 years old (adjusted incidence). The higher incidence in this age group is likely due to the higher morbidity of individuals ≥ 85 years old, which include generally higher co-morbidity burden and potentially more frailty and relative lower mobility [30].

The incidence of total hip joint X-rays also increased during the study period in people ≥ 65 years old. In comparison, during the same period, the incidence of surgically treated hip fractures and other surgical hip repairs did not change [31]. In 2004–05 it was reported that several fall-related injuries, such as fractures, sprains and strains, or bone conditions, such as osteoarthritis and osteoporosis, represented the main indications for which a plain X-ray was ordered [6]. Fracture risk is accentuated by the increase in osteoporosis prevalence in the older population and particularly in post-menopausal women. In 2012, it was estimated that 4.74 million Australians ≥ 50 years old had osteoporosis, osteopenia or poor bone health and that this number could increase by 31% in 2022 [32]. Because of the increase in osteoporosis in the older population, osteoporotic fractures represent a risk for this population with 32% of minimal trauma fractures considered to be osteoporotic hip fractures [33]. The difference in utilisation between sexes can also be due to a higher frailty incidence in women who have experienced menopause [34]. An increase in frailty and potentially in osteoporosis prevalence may be driving an increase in falls in this age category and therefore in plain X-ray utilisation for the diagnosis of fall-related injuries. The consistent increase in the utilisation of plain X-rays of extremities since 2009–10 indicates that this diagnostic tool remains essential in the health care management of individuals ≥ 65 years old.

The main limitation of this study is its reliance on publicly available data. Due to the limited granularity of the data used for this project, which is based on administrative service records, we could not examine the service indication or individual characteristics that may influence service use. This limits our ability to comment on the appropriateness of plain X-rays for the diagnoses of specific conditions and whether patient outcomes improved post-service. The size of age-sex groups varied, which could affect the level of difference in trends observed between the groups. Additionally, as X-rays conducted during hospitalisations or presentations to the ED can be claimed for the evaluation of outpatients, the distinction between services claimed in or outside of the hospital could not be made [15]. Therefore, this study provides an evaluation of Medicare subsidised services only and a limited evaluation of factors that influenced the changes in use of plain X-rays, rather than an exhaustive exploration of the possible factors. The advantage of the present approach lies in the great volume of data available for analysis, providing a strong basis to establish trends of X-ray utilisation.

## Conclusion

The crude utilisation of major plain X-rays has increased by 63% between 2009–10 and 2018–19 in Australia. The changes in X-ray use between 2009–10 and 2018–2019 varied by type of service, with the utilisation of plain X-rays of extremities and the hip joint increasing for most age-sex groups studied and lung X-ray utilisation increasing mostly in men ≥ 85 years old. Similarly, X-rays of extremities increased between 2009–10 and 2018–19, with differences between age-sex categories potentially explained by increases in prevalence of osteoporosis and frailty. The consistent and large increase in plain X-ray utilisation over the past ten years in Australia demonstrates that this diagnostic technique is still considered a standard approach in the care and health diagnosis of older citizens. The data presented demonstrates that unless policies change, there is potential for an important increase in plain X-rays utilisation by older Australians.

## Supplementary Information

**Additional file 1. **

**Additional file 2. **

## Data Availability

All data used herein was obtained from publicly available sources and relevant data providers have been acknowledged in the text, namely the Medicare statistics website and the Australian Bureau of Statistics (ABS).[1, 13] The access to the data is opened to everyone and no permission needs to be granted.Medicare statistics website: http://medicarestatistics.humanservices.gov.au/statistics/mbs_item.jsp, Australian Bureau of Statistics website: https://www.abs.gov.au/statistics#people

## Abbreviations

ABS: Australian Bureau of Statistics
CI: Confidence Interval
CT: Computed Tomography
GI: Gastro-intestinal
IR: Incidence Rate
IRR: Incidence Rate Ratio
MBS: Medicare Benefit Schedule
RACF: Residential Aged Care Facility
