# Supplementary material for: Trends in utilisation of plain X-rays by older Australians (2010–2019)
Source: BMC Geriatr. 2022 Feb 4;22:100. doi: 10.1186/s12877-022-02786-1 (PMC8817507; doi:10.1186/s12877-022-02786-1)
Supplement: Supplementary file 1 — Additional file 1. [file 12877_2022_2786_MOESM1_ESM.docx]

**Table S1** MBS codes investigated

| **Body area** | **Specific area** | **MBS items** | **Description and status during study period** | **Activity period covered** |
| --- | --- | --- | --- | --- |
|  |  |  |  |  |
| Chest | Lung | 58500 | Radiographic examination of thoracic region – Chest (lung fields) by direct radiography | 2009-10 to -2018-19, non-requested |
|  |  | 58502 | Radiographic examination of thoracic region – Chest (lung fields) by direct radiography | 2011-12 to 2018-19, non-requested, introduction of the item to differentiate services done with an equipment >10 years old |
|  |  | 58503 | Radiographic examination of thoracic region – Chest (lung fields) by direct radiography | 2009-10 to 2018-19, requested |
|  |  | 58505 | Radiographic examination of thoracic region – Chest (lung fields) by direct radiography | 2011-12 to 2018-19, requested, introduction of the item to differentiate services done with an equipment >10 years old |
|  |  |  |  |  |
|  | Thorax, thoracic | 58509 | Radiographic examination of thoracic region – Thoracic inlet or trachea | 2009-10 to 2018-19, requested |
|  |  | 58511 | Radiographic examination of thoracic region – Thoracic inlet or trachea | 2011-12 to 2018-19, requested, introduction of the item to differentiate services done with an equipment >10 years old |
|  | Rib | 58521 | Radiographic examination of thoracic region – Left ribs, right ribs of sternum | 2009-10 to 2018-19, requested |
|  |  | 58523 | Radiographic examination of thoracic region – Left ribs, right ribs or sternum | 2011-12 to 2018-19, requested, introduction of the item to differentiate services done with an equipment >10 years old |
|  |  |  |  |  |
| Chest (continue) | Rib  (continue) | 58524 | Radiographic examination of thoracic region – Left and right ribs, left ribs and sternum, or right ribs and sternum | 2009-10 to 2018-19, requested |
|  |  | 58526 | Radiographic examination of thoracic region – Left and right ribs, left ribs and sternum, or right ribs and sternum | 2011-12 to 2018-19 – requested, introduction of the item to differentiate services done with an equipment >10 years old |
|  |  | 58527 | Radiographic examination of thoracic region – Left ribs, right ribs and sternum | 2009-10 to 2018-19, requested |
|  |  | 58529 | Radiographic examination of thoracic region – Left ribs, right ribs and sternum | 2011-12 to 2018-19, requested, introduction of the item to differentiate services done with an equipment >10 years old |
|  |  |  |  |  |
| GI tract | Abdomen, abdominal | 58900 | Radiographic examination of alimentary tract and biliary system – Pain abdominal only | 2009-10 to 2018-19, non-requested |
|  |  | 58902 | Radiographic examination of alimentary tract and biliary system – Pain abdominal only | 2011-12 to 2018-19, non-requested, introduction of the item to differentiate services done with an equipment >10 years old |
|  |  | 58903 | Radiographic examination of alimentary tract and biliary system – Pain abdominal only | 2009-10 to 2018-19, requested |
|  |  | 58905 | Radiographic examination of alimentary tract and biliary system – Pain abdominal only | 2011-12 to 2018-19, requested, introduction of the item to differentiate services done with an equipment >10 years old |
|  |  |  |  |  |
|  |  |  |  |  |
|  |  |  |  |  |
| Extremities | Hand, wrist, elbow, humerus, forearm, shoulder, upper arm, clavicle | 57506 | Radiographic examination of extremities – Hand, wrist, forearm, elbow or humerus | 2009-10 to 2018-19, non-requested |
|  |  | 57509 | Radiographic examination of extremities – Hand, wrist, forearm, elbow or humerus | 2009-10 to 2018-19, requested |
|  |  | 57512 | Radiographic examination of extremities – Hand and wrist, or hand, wrist and forearm, or forearm and elbow, or elbow and humerus | 2009-10 to 2018-19, non-requested |
|  |  | 57515 | Radiographic examination of extremities – Hand and wrist, or hand, wrist and forearm, or forearm and elbow, or elbow and humerus | 2009-10 to 2018-19, requested |
|  |  | 57529 | Radiographic examination of extremities – Hand, wrist, forearm, elbow or humerus | 2011-12 to 2018-19, non -requested, introduction of the item to differentiate services done with an equipment >10 years old |
|  |  | 57530 | Radiographic examination of extremities – Hand, wrist, forearm, elbow or humerus | 2011-12 to 2018-19, requested, introduction of the item to differentiate services done with an equipment >10 years old |
|  |  | 57532 | Radiographic examination of extremities – Hand and wrist, or hand, wrist and forearm, or forearm and elbow, or elbow and humerus | 2011-12 to 2018-19, non -requested, introduction of the item to differentiate services done with an equipment >10 years old |
|  |  | 57533 | Radiographic examination of extremities – Hand and wrist, or hand, wrist and forearm, or forearm and elbow, or elbow and humerus | 2011-12 to 2018-19, requested, introduction of the item to differentiate services done with an equipment >10 years old |
|  |  |  |  |  |
| Extremities (continue) | Hand, wrist, elbow, humerus, forearm, shoulder, upper arm, clavicle (continue) | 57521 | Radiographic examination of extremities – Foot, ankle, leg, knee or femur | 2009-10 to 2018-19, requested |
|  |  | 57700 | Radiographic examination of shoulder or pelvis – Shoulder or scapula | 2009-10 to 2018-19, non-requested |
|  |  | 57702 | Radiographic examination of shoulder or pelvis – Shoulder or scapula | 2011-12 to 2018-19, non-requested, introduction of the item to differentiate services done with an equipment >10 years old |
|  |  | 57703 | Radiographic examination of shoulder or pelvis – Shoulder or scapula | 2009-10 to 2018-19, requested |
|  |  | 57705 | Radiographic examination of shoulder or pelvis – Shoulder or scapula | 2011-12 to 2018-19, introduction of the item to differentiate services done with an equipment >10 years old |
|  |  | 57706 | Radiographic examination of extremities – Clavicle | 2009-10 to 2018-19, non-requested |
|  |  | 57708 | Radiographic examination of extremities – Clavicle | 2011-12 to 2018-19, non-requested, introduction of the item to differentiate services done with an equipment >10 years old |
|  |  | 57709 | Radiographic examination of extremities – Clavicle | 2009-10 to 2018-19, requested |
|  |  | 57711 | Radiographic examination of extremities – Clavicle | 2011-12 to 2018-19, non-requested, introduction of the item to differentiate services done with an equipment >10 years old |
|  |  |  |  |  |
| Extremities (continue) | Foot, ankle, leg, femur, knee | 57518 | Radiographic examination of extremities – Foot, ankle, leg, knee, or femur | 2009-10 to 2018-19, non-requested |
|  |  | 57521 | Radiographic examination of extremities – Foot, ankle, leg, knee, or femur | 2009-10 to 2018-19, requested |
|  |  | 57522 | Radiographic examination of extremities – Knee, bulk billing incentive | 2018-19, non-requested, introduction of item to differentiate service conducted on knee only |
|  |  | 57523 | Radiographic examination of extremities – Knee, bulk billing incentive | 2018-19, requested, introduction of item to differentiate service conducted on knee only |
|  |  | 57524 | Radiographic examination of extremities – Foot and ankle, or ankle and leg, or leg and knee, or knee and femur | 2009-10 to 2018-19, non-requested |
|  |  | 57527 | Radiographic examination of extremities – Foot and ankle, or ankle and leg, or leg and knee, or knee and femur | 2009-10 to 2018-19, requested |
|  |  | 57535 | Radiographic examination of extremities – Foot, ankle, leg, knee or femur | 2011-12 to 2018-19, non-requested, introduction of the item to differentiate services done with an equipment >10 years old |
|  |  | 57536 | Radiographic examination of extremities – Foot, ankle, leg, knee or femur | 2011-12 to 2018-19, requested, introduction of the item to differentiate services done with an equipment >10 years old |
|  |  | 57537 | Radiographic examination of extremities – Knee, bulk billing incentive (lower level of subsidisation) | 2018-19, non-requested, introduction of item to differentiate service conducted on knee only |
|  |  |  |  |  |
| Extremities (continue) | Foot, ankle, leg, femur, knee (continue) | 57538 | Radiographic examination of extremities – Foot and ankle, or ankle and leg, or leg and knee, or knee and femur | 2011-12 to 2018-19, non-requested, introduction of the item to differentiate services done with an equipment >10 years old |
|  |  | 57539 | Radiographic examination of extremities – Foot and ankle, or ankle and leg, or leg and knee, or knee and femur | 2011-12 to 2018-19, requested, introduction of the item to differentiate services done with an equipment >10 years old |
|  |  | 57540 | Radiographic examination of extremities – Knee, bulk billing incentive (lower level of subsidisation) | 2018-19, non-requested, introduction of item to differentiate service conducted on knee only |
| Hip and pelvis | Hip joint | 57712 | Radiographic examination of shoulder or pelvis – Hip joint | 2009-10 to 2018-19, requested |
|  |  | 57714 | Radiographic examination of shoulder or pelvis – Hip joint | 2011-12 to 2018-19, introduction of the item to differentiate services done with an equipment >10 years old |
|  | Pelvic girdle | 57715 | Radiographic examination of shoulder or pelvis – Pelvic girdle | 2009-10 to 2018-19, requested |
|  |  | 57717 | Radiographic examination of shoulder or pelvis – Pelvic girdle | 2011-12 to 2018-19, requested, introduction of the item to differentiate services done with an equipment >10 years old |

**Abbreviations:** GI= gastro-intestinal.

**Note:** A range of items started in the financial year 2011-12 to differentiate services conducted with equipment over 10 years old. Several items were introduced in the 2018-19 financial year to separate examinations of the knee on its own, encouraging bulk billing for inpatient examinations at several levels of subsidisation. Services are noted as requested or non-requested. A requested service was ordered by a general practitioner.

**Source:** All available Medicare Benefits Schedule Book Category 5 since 2009 and the Australian Government Services Australia website (http://medicarestatistics.humanservices.gov.au/statistics/mbs_item.jsp).

2009-10 (http://www.mbsonline.gov.au/internet/mbsonline/publishing.nsf/Content/B55546F33798EF39CA257CD00081F202/$File/CACHE_DUVIE=2a813ed6b688d587f724e6e551f76b67/200911-MBS.pdf)

2010-11 (http://www.mbsonline.gov.au/internet/mbsonline/publishing.nsf/Content/01EB18082211C495CA257CCF00051C1B/$File/201101-Cat%205.pdf)

2014-15

(http://www.mbsonline.gov.au/internet/mbsonline/publishing.nsf/Content/85A357DC1CD8BA24CA257D6600087FAD/$File/201407-Cat5.pdf),

2015-16 (http://www.mbsonline.gov.au/internet/mbsonline/publishing.nsf/Content/9B067E48C350D16BCA257DAA007C462D/$File/CACHE_DUVIE=ef3c5693a5395a0dab8b65aaea6f952c/201501-Cat5.pdf)

2017-18 (http://www.mbsonline.gov.au/internet/mbsonline/publishing.nsf/content/ED28842309B2CD13CA2580F7008294BE/$File/CACHE_DUVIE=4d538aa2147f5397233fd869358a5a49/201705-Cat5.pdf)

2018-19 (http://www.mbsonline.gov.au/internet/mbsonline/publishing.nsf/Content/95179E0FC752A061CA2581EC000F4188/$File/CACHE_DUVIE=78a963fa82a777101e57e27fa82123e5/201801-Cat5.pdf)
