# Supplementary material for: Trends in utilisation of plain X-rays by older Australians (2010–2019)
Source: BMC Geriatr. 2022 Feb 4;22:100. doi: 10.1186/s12877-022-02786-1 (PMC8817507; doi:10.1186/s12877-022-02786-1)
Supplement: Supplementary file 2 — Additional file 2. [file 12877_2022_2786_MOESM2_ESM.docx]

**Supplementary Table 2:** Number of Australians ≥65 years old between 2010 and 2019 by gender and age group as provided by the Australian Bureau of Statistics.

|  | **2010** | **2011** | **2012** | **2013** | **2014** | **2015** | **2016** | **2017** | **2018** | **2019** |
| --- | --- | --- | --- | --- | --- | --- | --- | --- | --- | --- |
|  |  |  |  |  |  |  |  |  |  |  |
| **Men** |  |  |  |  |  |  |  |  |  |  |
| 65-74 | 761,288 | 822,878 | 813,549 | 920,511 | 956,825 | 989,503 | 1,025,951 | 1,057,029 | 1,089,381 | 1,114,146 |
| 75-84 | 439,404 | 448,527 | 448,983 | 470,988 | 485,668 | 495,902 | 510,657 | 531,605 | 552,182 | 579,485 |
| ≥85 | 127,528 | 138,934 | 139,070 | 156,490 | 164,903 | 171,882 | 179,238 | 185,066 | 190,647 | 198,110 |
| Total ≥65 | 1,328,220 | 1,410,339 | 1,419,602 | 1,547,989 | 1,607,396 | 1,657,287 | 1,715,846 | 1,773,700 | 1,832,210 | 1,891,741 |
|  |  |  |  |  |  |  |  |  |  |  |
| **Women** |  |  |  |  |  |  |  |  |  |  |
| 65-74 | 793,853 | 848,781 | 850,382 | 942,751 | 982,490 | 1,017,187 | 1,057,318 | 1,094,545 | 1,134,663 | 1,168,531 |
| 75-84 | 544,632 | 553,046 | 553,390 | 564,323 | 574,672 | 582,767 | 595,646 | 614,740 | 633,686 | 660,313 |
| ≥85 | 247,631 | 264,373 | 264,537 | 283,105 | 291,630 | 297,063 | 303,441 | 307,857 | 312,416 | 317,594 |
| Total ≥65 | 1,586,116 | 1,666,200 | 1,668,309 | 1,790,179 | 1,848,792 | 1,897,017 | 1,956,405 | 2,017,142 | 2,080,765 | 2,146,438 |
|  |  |  |  |  |  |  |  |  |  |  |
| **Persons** |  |  |  |  |  |  |  |  |  |  |
| 65-74 | 1,555,141 | 1,671,659 | 1,681,931 | 1,863,262 | 1,939,315 | 2,006,690 | 2,083,269 | 2,251,574 | 2,224,044 | 2,282,677 |
| 75-84 | 984,036 | 1,001,573 | 1,002,373 | 1,035,311 | 1,060,340 | 1,078,669 | 1,106,303 | 1,146,345 | 1,185,868 | 1,239,798 |
| ≥85 | 375,159 | 403,307 | 403,607 | 439,595 | 456,533 | 468,945 | 482,679 | 492,923 | 503,063 | 515,704 |
| Total ≥65 | 2,914,336 | 3,076,539 | 3,087,911 | 3,338,168 | 3,456,188 | 3,554,304 | 3,790,842 | 3,790,842 | 3,912,975 | 4,038,179 |
|  |  |  |  |  |  |  |  |  |  |  |

**Source:** The Australian Bureau of Statistics (https://www.abs.gov.au/statistics/people/population).
